# Copenhagen Psychosocial Questionnaire - A validation study using the Job Demand-Resources model

**DOI:** 10.1371/journal.pone.0196450

**Published:** 2018-04-30

**Authors:** Hanne Berthelsen, Jari J. Hakanen, Hugo Westerlund

**Affiliations:** 1 Centre for Work Life and Evaluation Studies, Malmö University, Malmö, Sweden; 2 Faculty of Odontology, Malmö University, Malmö, Sweden; 3 Finnish Institute of Occupational Health, Helsinki, Finland; 4 Helsinki Collegium for Advanced Studies, University of Helsinki, Helsinki, Finland; 5 Stress Research Institute, Stockholm University, Stockholm, Sweden; Public Library of Science, UNITED KINGDOM

## Abstract

**Aim:**

This study aims at investigating the nomological validity of the Copenhagen Psychosocial Questionnaire (COPSOQ II) by using an extension of the Job Demands-Resources (JD-R) model with aspects of work ability as outcome.

**Material and methods:**

The study design is cross-sectional. All staff working at public dental organizations in four regions of Sweden were invited to complete an electronic questionnaire (75% response rate, n = 1345). The questionnaire was based on COPSOQ II scales, the Utrecht Work Engagement scale, and the one-item Work Ability Score in combination with a proprietary item. The data was analysed by Structural Equation Modelling.

**Results:**

This study contributed to the literature by showing that: A) The scale characteristics were satisfactory and the construct validity of COPSOQ instrument could be integrated in the JD-R framework; B) Job resources arising from leadership may be a driver of the two processes included in the JD-R model; and C) Both the health impairment and motivational processes were associated with WA, and the results suggested that leadership may impact WA, in particularly by securing task resources.

**Conclusion:**

In conclusion, the nomological validity of COPSOQ was supported as the JD-R model-can be operationalized by the instrument. This may be helpful for transferral of complex survey results and work life theories to practitioners in the field.

## Introduction

The Copenhagen Psychosocial Questionnaire (COPSOQ) is one of a few research-based instruments that have been developed for use at workplaces as well as for research purposes [1]. The instrument is internationally recognized as a risk assessment tool by both the International Labour Organization and the World Health Organization [1, 2] and is used in workplace surveys worldwide for work environment development and follow-up of organizational changes[3, 4].

COPSOQ is a generic, theory-based questionnaire covering a broad range of aspects of the psychosocial working environment rather than being linked to a specific theoretical framework [5, 6]. The instrument covers central dimensions of the seven theories of psychosocial factors at work [5], which were identified as most influential by Kompier [7] in 2003: The Job Characteristics Model, The Michigan Organization Stress Model, The Job Demands–Control Model, The Sociotechnical Approach, The Action-Theoretical Approach, The Effort–Reward Imbalance Model and, The Vitamin Model.

COPSOQ validation studies have been conducted in a number of countries (see e.g. [6, 8–16]) and various aspects of the reliability and validity have been tested. Among these, test-retest reliability [14, 16, 17], minimally important score differences [18], differential item functioning and differential item effect [19], and criterion-related validity in relation to e.g. different measures of sickness absence [6, 20]. Construct validity has been corroborated through analyses of inter-scale-correlations, and also in relation to other instruments measuring corresponding constructs (see. e.g.[14, 16]). So far, however, no study has tested the concurrent validity of COPSOQ scales in one comprehensive, theoretically based model. In this study, we focus on aspects of WA as an outcome in relation to an extended JD-R model.

Work ability (WA) is important for the individual employee, the workplaces and for society. WA is a multifaceted construct which in epidemiological research typically consists of the worker’s self-assessed ability to work now and in the near future with respect to work demands, health and mental resources [21]. Associations between COPSOQ scales and WA have been demonstrated in a number of recent studies [10, 22–28]. Results from these studies demonstrate negative associations between work ability and *Quantitative & Emotional Demands* [22, 24, 25], *Role Conflicts* [22] *Work Family Conflict* [24] *as well as Stress* [22–24], *Burnout* [22] and *Sleeping Troubles* [22, 23]. In contrast, positive associations with work ability have been reported in relation to *Influence* [24, 25], *Possibilities for Development* [22, 25], *Meaning in Work and Role Clarity* [22], *Quality in Leadership* [22, 25], *Social Support* [23–25], *Social Community at Work* [22, 23, 25], *Job Satisfaction* [22, 24, 26], *Justice and Respect* [22] and *General Health* [23]. Our operationalization in the present study comprises a combination of self-rated general health [6], current global WA [21, 29] and expected future health-related WA in the present occupation.

In recent years, the Job Demands-Resources (JD-R) model has become one of the most influential stress and motivation models in work and organizational psychology [30, 31]. It has been validated in many cross-sectional studies (e.g. [32, 33]) and also longitudinally [34]. The model is comprehensive and drawing on classical job satisfaction theories in addition to previous work environment models [30, 35]. This makes it especially suitable for use at workplaces, where a holistic approach is needed.

The basic assumption of the JD-R model is that all psychosocial work characteristics can be categorized into demands and resources [32, 36]. *Job demands* refer to physical, psychological, social, or organizational aspects of a job that require sustained physical and/or psychological effort (e.g. workload, role conflicts), whereas *job resources* (e.g. social support, job autonomy) refer to aspects of the job that may reduce job demands and the associated physiological and psychological costs, are functional in achieving work goals, and stimulate personal growth, learning, and development. The model posits that high job demands may trigger a health impairment process (leading to strain and burnout and further to ill-health), whereas high job resources are “energizers” initiating a motivational process leading to positive attitudes towards work and positive behaviours and may also reduce strain symptoms. However, not all resources and demands interact equally in relation to strain [35], and also it has become apparent that the complexity of the concepts is higher than assumed in the early days of the JD-R model [37]. The role of hindrance and challenge demands have become the subject of research and there might be a need of such distinctions even in relation to job resources [37, 38].

Bakker and Demerouti have proposed a division between job resources arising from organizational, interpersonal, and task level [39]. While task resources was regarded as most important for motivational outcomes in the job characteristics theory [40] a shift in work life research has been seen during recent years [4]. Today, work-life interpersonal relations are considered highly relevant as for example argued by Grant and Parker [41]. This shift is reflected by the COPSOQ II scales, which comprises job factors, relational factors, leadership and climatic factors in addition to a number of health-related as well as motivational outcomes.

Formal leaders have a crucial role for employee wellbeing and health as they can affect working conditions such as amount of assignments, role clarity and influence as well as the social environment [42]. Accordingly, job resources provided by leaders may be perceived as antecedents for task and interpersonal resources. In relation to the JD-R model, Schaufeli has recently pointed to this specific role of engaging leadership [43]. His findings from analyses based on an extended JD-R model indicate that engaging leadership affects wellbeing of the employees indirectly via the impact on job demands, but in particular on job resources.

Simultaneously testing associations between the entire COPSOQ instrument and aspects of WA in a nomological framework will go one step further than previous validity studies on the instrument. Besides, it will add to an overview, which in particularly may be helpful for transferral of complex methods and theories from research to practice.

In the present study, we applied an extended JD-R model based on domains suggested by results from previous validation studies of COPSOQ II [6, 8, 10]. We aimed at testing the concurrent validity of the entire COPSOQ instrument with aspects of WA as outcome using an extended JD-R model with leadership resources as an antecedent to job demands and two kinds of job resources: task resources and interpersonal resources.

## Materials and methods

### Questionnaire development and data collection

In 2003 a team of Danish and Swedish researchers (Arvidsson, Johansson, Kolstrup & Pousette) made a first translation of the Danish version of COPSOQ into Swedish and this work was updated by Ektor-Andersen until a final version was established in 2007 [44]. Even though scales from this version have been used in Sweden in a number of research projects since then, the Swedish version of the instrument has not previously been subject for a validation study. The validation process included a back-translation of the existing Swedish version of COPSOQ II into English, a systematic evaluation process and five rounds of cognitive interviews using a think aloud procedure with additional probing[45–47]. Interviews were conducted with 26 informants selected to achieve variation in gender, age, region of residence, and occupation. The overall purpose was to develop the formulations of the items by identifying potential problems in the questionnaire and clarifying how informants understood key concepts at an early stage of the process as suggested by Willis [48, 49]. Based on the findings from the back-translation and the interviews, the Swedish version of COPSOQ was revised and tested on new rounds of informants until well-functioning formulations conceptually equivalency with the English version was achieved. The initial steps of the validation process corroborated face and content validity of the items (further details have been published elsewhere [45–47]). The Utrecht Work Engagement scale [50, 51], the one-item Work Ability Score [29, 52] and other additional items were tested similarly.

The data for the present study was collected from May 2014 to January 2015. All staff employed at the Public Dental Health Service in four regions of Sweden (N = 1782) received an email with a personal login and password to an online questionnaire and after two reminders 1345 respondents had replied, providing a response rate of 75% (ranging from 71%–81% among the regions). Employees on long-term sickness absence or parental leave were excluded from the sampling frame as presence at the workplace is required for the questionnaire to be relevant to fill in. This has probably led to an overestimation of the true level of work ability for the total work force and a risk of underestimation of the strength of the associations in the model tested as research show that e.g. self-reported sickness absence predicts future reduced work ability [53].

Respondents were on average almost three years older than the non-respondents (*p*≤0.001). Pearson chi-square tests revealed that the response rate was higher for managers than for other employees (91.8% vs. 73.8%, *p*≤0.001). The response rates also differed between occupational groups: dentists having the lowest (67.8%) and employees with educational backgrounds outside dentistry the highest (84.2% (*p*≤0.001)).

### Study population

The study sample (Table 1) comprises primarily Swedish-born women, and the mean age of the total sample was 48.5 (SD 11.3) years. The respondents worked on average 36.9 (SD 6.0) hours per week, had worked 17.3 (SD 13.8) years in the same organization and almost all had a permanent position (98.1%).

**Table 1:** Demographic distribution of the respondents in percentages.

Category	Description	Percentage
Gender	Women	89.8
	Men	10.2
Age group	≤29	8.2
	30–39	16.2
	40–49	22.8
	50–59	34.3
	≥60	18.6
Occupation	Dental nurse	50.9
	Dental hygienist	17.5
	General dental practitioner	21.3
	Specialized dental practitioner	4.1
	Dental technician	1.4
	Other education	4.8
Position	Non-managerial staff	88.6
	Managerial staff	11.4

The Swedish public dental sector comprises large regional organizations including service facilities, administration in addition to general and specialized dental clinics. The sector has often been described as influenced by New Public Management, in particularly regarding management by objectives with an emphasis on quantitative measures of productivity (e.g. [54–58]). There is a widespread belief among senior management of the regional dental organizations that large clinics with a formal management structure is an advantage [59]. While the objectives in economic terms largely are given from the organization, the local leadership may differ within the organization [59]. Based on the context it therefore seems likely that first line managers have more opportunities for influencing interpersonal relations and task resources than job demands.

### Measures

In general, COPSOQ items have five response options on Likert-type scales, for example from never to always or from a very low to a very high extent. For the analyses, items were scored 100, 75, 50, 25, 0, and scale scores calculated as the mean of the items for each scale, including only those respondents who had answered at least half of the questions included in the scale [6].

Work engagement was measured by the nine-item version of the Utrecht work engagement scale [50, 51]. Each item had seven response options on a Likert-type scale ranging from 0 = never to 6 = always. The scale score was computed as mean of item scores.

WA was measured by three items A) self-assessment of current global WA as compared with the lifetime best WA on a scale from 0–10 [52], B) general self-rated health from COPSOQ II [6] and C) prospective health-related WA by asking: “Considering your health, do you believe that you can work in your current job even in two years?” with the response alternatives: No, hardly; maybe; yes, probably and scored 0, 50, 100.

Multiple indicators for each latent variable were used in the tested models. Leadership Resources were indicated by five scales, Interpersonal Resources by three scales, Task Resources by three scales and one additional single item, Job Demands by five scales, Strain Indicators by three scales, Positive Work Attitudes by three scales and WA was indicated by three single items. We chose to exclude the COPSOQ scales for Meaning in Work and for Vertical Trust from the analyses due to a conceptual overlap and high shared variance with Work Engagement and Horizontal Trust, respectively.

### Analyses

Data analyses were conducted using IBM SPSS and AMOS version 23 [60]. More than 15% of the respondents choosing the lowest or highest response options was considered evidence of a floor or ceiling effect, respectively [61].

A number of indices were used to examine the overall fit of the hypothesized and alternative models to the data: χ^2^ test and Root Mean Square Error of Approximation (RMSEA) as absolute goodness-of-fit indices. RMSEA values below 0.05 indicate good fit, 0.06–0.08 reasonable fit, 0.08–0.10 mediocre fit, and >0.10 poor fit [62–64]. In addition, relative goodness-of-fit indices were investigated: the Comparative Fit Index (CFI) and Tucker-Lewis Index (TLI). The classical criterion for these two indices suggests that values over 0.90 and even over 0.95 indicate a good fit [62]. The fit of nested models was compared by testing the significant changes in the χ^2^ values and with nested models we used the Akaike Information Criterion (AIC) to compare the models; the smaller the value of AIC, the better fitting the model is [65]. The statistical significance of the indirect effects was tested using bootstrapping procedures (5000 bootstrap samples, 95% two-sided CI).

### Ethics

The study was approved by the Regional Ethics Board in Southern Sweden (Dnr. 2013/256 & 2013/505) and informed consent was obtained from all individual participants included in the study.

## Results

### Scale characteristics

Scale characteristics included in the Swedish COPSOQ II version and other variables included in the present study are presented in Table 2. The internal consistencies were above 0.70 for all scales except for *Role Conflicts* (0.65). The proportion of internally missing values for the scales was below 2% except for the two items asking whether the employees withhold information from the management, and vice versa, as well as one item asking whether the nearest superior is good at handling conflicts (2.9–3.3% missing values). The scale for *Meaning in Work* had a high ceiling effect (20.2%) and a high mean score (80.7 st.dev. 15.5). The scales for *Role Clarity* and *Social Community at Work* also showed some ceiling effect (15.2%–17.3%), while *Sleeping Troubles* and *Work-Family Conflict* had a corresponding floor effect (15.8–16.9%). Correlations between all study variables are presented in Table 3.

**Table 2:** Psychometric characteristics of the scales and the hypothesized higher order factors.

Hypothesized Factor	Factor loading	Scale (number of items)	Mean	St.Dev.	Missing pct. (scale)	Floor pct.	Ceiling pct.	Cronbach’s alpha
**Demands**	0.66	Quantitative Demands (4)*	45.40	17.84	0.00	1.00	0.30	0.83
	0.56	Work Pace (3)*	70.20	19.04	0.07	0.20	10.10	0.87
	0.53	Emotional Demands (4)*	53.54	18.33	0.00	0.70	0.60	0.80
	0.58	Role Conflicts (4)*	34.88	16.63	0.30	3.00	0.00	0.65
	0.71	Work Family Conflict (4)*	32.45	26.68	0.22	16.90	1.90	0.83
**Task Resources**	0.53	Influence (4)*	46.06	17.69	0.00	0.60	0.30	0.74
	0.79	Possibilities for Development *(4)	72.22	15.50	0.00	0.10	5.10	0.73
	0.55	Variation (1)*	70.81	21.13	0.30	1.10	20.20	–
	0.53	Role Clarity (3)*	79.97	14.37	0.00	0.10	15.20	0.72
**Interpersonal Resources**	0.74	Social Support Colleagues (3)*	68.77	16.04	0.22	0.10	1.90	0.71
	0.82	Social Community at Work (3)*	79.73	14.18	0.07	0.10	17.30	0.80
	0.56	Horizontal Trust (3)*	73.12	17.99	1.93	0.00	11.40	0.78
**Leadership Resources**	0.81	Quality Leadership (4) *	62.59	22.05	1.12	1.60	5.90	0.90
	0.75	Social Support Superior (3)*	68.46	20.26	0.74	0.70	3.60	0.82
	0.87	Recognition (3)*	67.67	21.10	0.45	0.70	8.70	0.85
	0.81	Predictability (2)*	65.26	19.44	0.15	0.70	6.40	0.71
	0.88	Organizational Justice (4) *	61.59	18.25	0.67	0.10	2.40	0.84
		Vertical Trust (4)*	72.22	17.33	0.37	0.10	6.90	0.78
**Strain Symptoms**	0.91	Stress (4)*	34.29	23.90	0.22	8.80	1.30	0.91
	0.92	Burnout (4)*	37.87	23.66	0.15	5.10	1.80	0.91
	0.74	Sleep (4)*	30.35	24.73	0.15	15.80	1.30	0.90
**Positive Work Attitudes**	0.84	Job Satisfaction (4)*	69.04	17.81	0.22	0.30	5.70	0.84
	0.83	Commitment Work (3)*	71.00	19.74	0.22	0.20	10.90	0.72
	0.71	Work Engagement (9)** (Scale 0–6)	4.28	0.93	0.67	0.07	2.10	0.94
**Health-related work ability**	0.66	General Health (1)*	62.19	21.04	1.64	1.00	9.70	–
	0.74	Work ability score (1)*** (Scale 0–10)	8.27	1.48	0.22	0.07	20.56	–
	0.44	Prospective health-related work ability (1) ****	91.64	21.97	0.37	2.69	85.97	–
		Meaning in Work (3)*	80.69	15.51	0.00	0.10	20.20	0.77

* COPSOQ scales (theoretical range 0–100)

** Utrecht Work Engagement Scale (theoretical range 0–6)

*** Work ability score (single item scale 0–10)

**** Proprietary item (theoretical range 0–100)

**Table 3:** Correlations between the study variables (n = 1345).

Variables	1.	2.	3.	4.	5.	6.	7.	8.	9.	10.	11.	12.	13.	14.	15.	16.	17.	18.	19.	20.	21.	22.	23.	24.	25.	26.	27.	28.
**1. Quantitative Demands**	-																											
**2. Work Pace**	.44	-																										
**3. Emotional Demands**	.35	.36	-																									
**4. Role Conflicts**	.42	.28	.39	-																								
**5.Work Family Conflict**	.48	.34	.32	.38	-																							
**6. Influence**	-.07	-.16	.03	-.04	-.11	-																						
**7. Possibilities for Development**	-.01	.05	.15	-.08	-.05	.45	-																					
**8. Variation**	-.02	.01	.08	-.03	-.03	.35	.42	-																				
**9. Role Clarity**	-.23	.01	-.10	-.36	-.18	.16	.41	.12	-																			
**10. Social Support Colleagues**	-.20	-.14	-.06	-.26	-.23	.31	.32	.19	.32	-																		
**11. Social Community at Work**	-.17	-.08	-.12	-.33	-.21	.23	.28	.19	.32	.61	-																	
**12. Horizontal Trust**	-.10	-.05	-.08	-.34	-.22	.14	.18	.14	.25	.37	.47	-																
**13. Quality Leadership**	-.19	-.14	-.07	-.25	-.19	.30	.38	.23	.40	.39	.41	.34	-															
**14. Social Support Superior**	-.18	-.14	-.08	-.22	-.16	.28	.38	.20	.39	.44	.38	.28	.76	-														
**15. Recognition**	-.20	-.17	-.11	-.29	-.25	.40	.48	.27	.42	.47	.49	.36	.68	.69	-													
**16. Predictability**	-.24	-.16	-.12	-.32	-.22	.33	.42	.26	.48	.41	.43	.35	.66	.58	.69	-												
**17. Organizational Justice**	-.21	-.18	-.10	-.31	-.23	.35	.43	.27	.44	.43	.47	.39	.75	.65	.76	.70	-											
**18. Vertical Trust**	-.19	-.12	-.09	-.35	-.23	.27	.39	.26	.43	.37	.43	.48	.67	.60	.69	.65	.78	-										
**19. Stress**	.41	.38	.35	.36	.60	-.19	-.13	-.14	-.19	-.30	-.35	-.28	-.29	-.27	-.37	-.32	-.35	-.32	-									
**20. Burnout**	.39	.34	.35	.35	.56	-.22	-.20	-.18	-.23	-.31	-.34	-.25	-.30	-.29	-.38	-.35	-.36	-.34	.83	-								
**21. Sleep**	.24	.24	.24	.25	.41	-.16	-.18	-.15	-.18	-.26	-.28	-.19	-.22	-.23	-.31	-.25	-.27	-.24	.67	.69	-							
**22. Job Satisfaction**	-.27	-.21	-.15	-.34	-.31	.37	.55	.37	.43	.41	.44	.30	.52	.50	.61	.56	.60	.57	-.45	-.49	-.35	-						
**23. Commitment Work**	-.26	-.19	-.12	-.36	-.35	.33	.48	.33	.42	.44	.51	.35	.58	.50	.65	.58	.61	.58	-.44	-.47	-.37	.70	-					
**24. Work Engagement**	-.18	-.05	-.02	-.24	-.23	.33	.50	.38	.36	.32	.33	.25	.38	.35	.43	.42	.41	.40	-.35	-.40	-.29	.59	.61	-				
**25. General Health**	-.16	-.11	-.05	-.13	-.26	.18	.26	.19	.15	.23	.21	.21	.24	.22	.27	.24	.26	.24	-.39	-.44	-.38	.34	.29	.34	-			
**26. Work ability score**	-.19	-.07	-.10	-.22	-.27	.13	.25	.16	.25	.25	.28	.18	.24	.24	.28	.25	.25	.26	-.41	-.43	-.38	.37	.34	.43	.50	-		
**27. Prospective health-related work ability**	-.13	-.10	-.08	-.13	-.16	.16	.17	.12	.13	.17	.18	.14	.20	.19	.21	.20	.20	.18	-.23	-.26	-.27	.27	.27	.29	.25	.35	-	
**28. Meaning in Work**	-.15	-.02	-.01	-.25	-.17	.31	.66	.37	.50	.32	.39	.24	.38	.35	.48	.43	.43	.40	-.27	-.32	-.28	.59	.59	.68	.29	.35	.25	-

Correlations │ ≥ .07 │ are statistically significant, p < .01; correlations │ = .06 │ are statistically significant, p < .05; correlations │ ≤.05 │ are not statistically significant.

### Relationships between the COPSOQ scales and work ability

**Fig 1:**
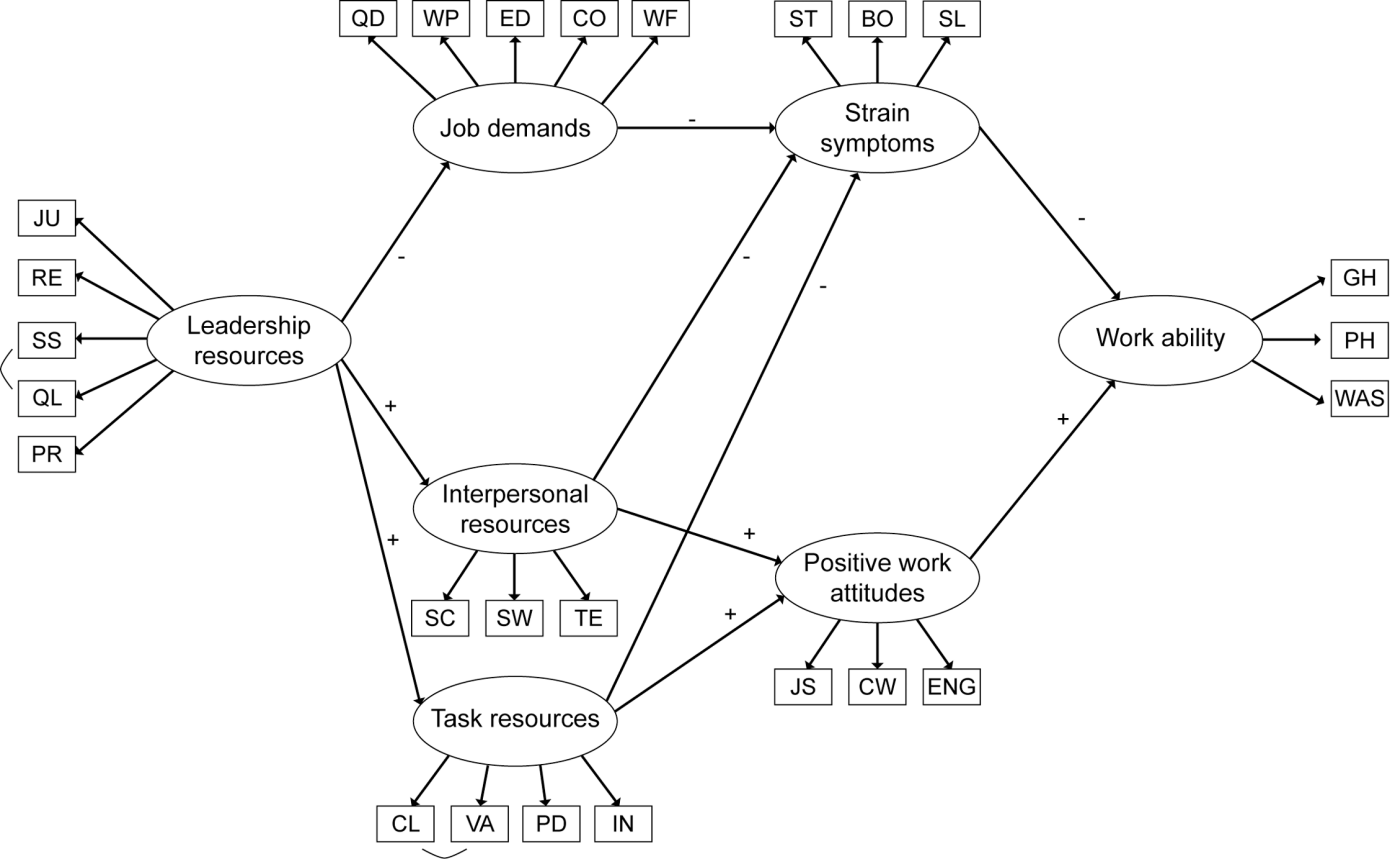
Overview of the study model.

First we tested the Confirmatory Factor Analytic (CFA) measurement model which specifies the pattern by which each measure loads on a particular factor (p6 in [66]) The CFA model presented an acceptable fit to the data (χ^2^(276) = 1552.00; CFI = .92; TLI = .91; RMSEA = .061). The initial model was respecified to allow error covariance between *Social Support from Superior* and *Quality of Leadership*, *and also between Variation* and *Role Clarity* based on the inspection of the modification indices and their conceptual interrelatedness. Items belonging to *Social Support from Superior* and *Quality of Leadership* inquires about the personal relation to the nearest superior, in contrast to items on e.g. *Organizational Justice*, which are based on a shift of referent addressing the climate at work [47]. Role clarity addresses issues such as the extent to which the employee knows exactly his/her areas of responsibility, which naturally is related to how much variation the job offers. The scales and items loaded on the factors as expected, and factor loadings ranged from .44–.92 (Table 2). Next we tested the proposed fully mediated model A (Fig 1) against alternative models.

**Fig 2:**
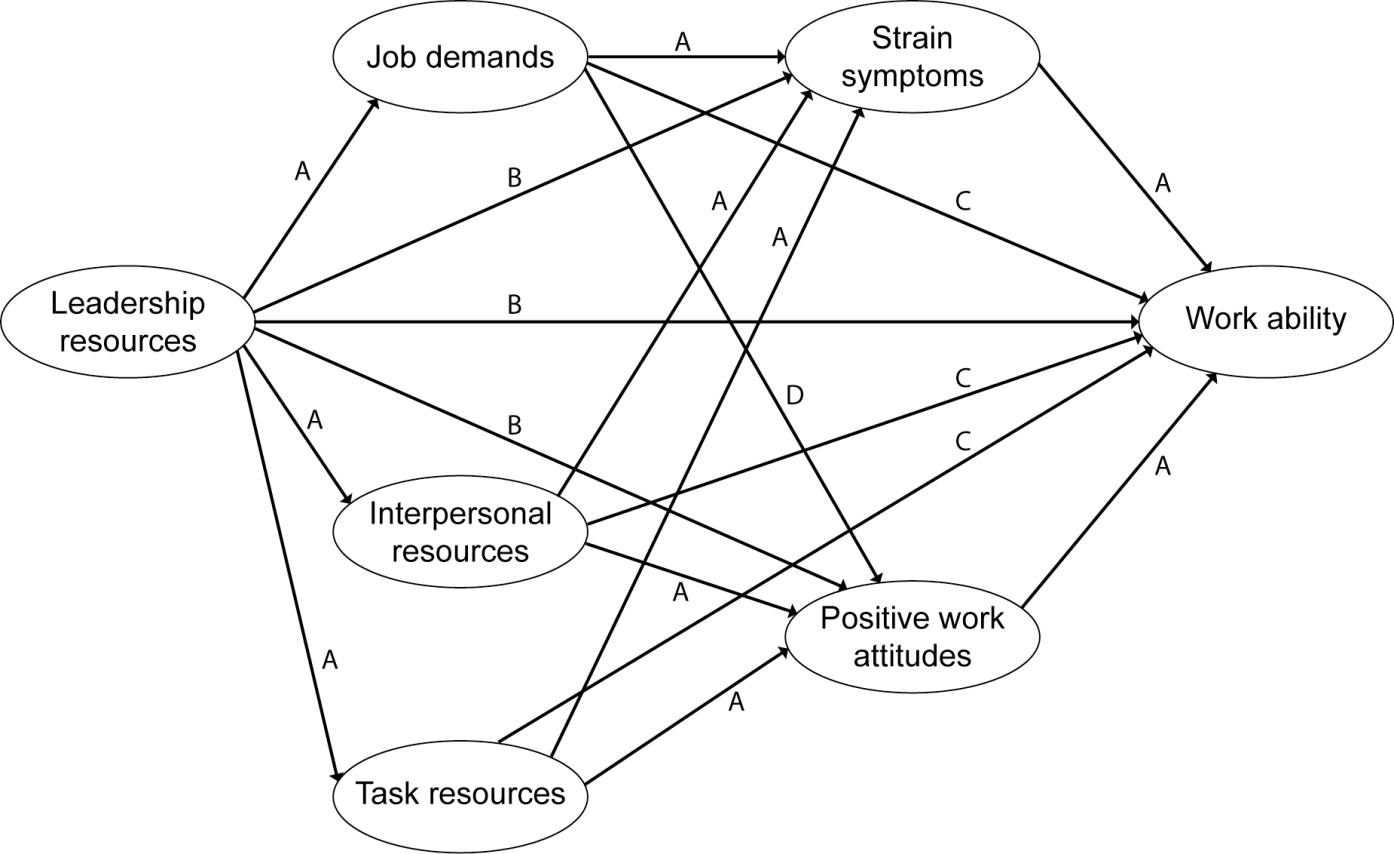
Overview of model A-D.

Model A included paths from Leadership to Job Demands, and Task and Interpersonal Resources (first set of mediators), which in turn were related to Strain Symptoms and Positive Work Attitudes (second set of mediators), and finally these latent variables were related to WA. The partially mediated model B included the paths in model A plus the direct paths from Leadership to Strain Symptoms and Positive Work Attitudes and to WA. Another partially mediated model C was like model B and additionally included direct paths from Job Demands, and Task and Interpersonal Resources to WA. Finally in model D, although not expected in the JD-R model, a path from Job Demands to Positive Work Attitudes was added in the model, as previous studies indicate that job demands may also be related to positive work attitudes [34, 58, 67]. An overview of all models is presented in Fig 2.

**Fig 3:**
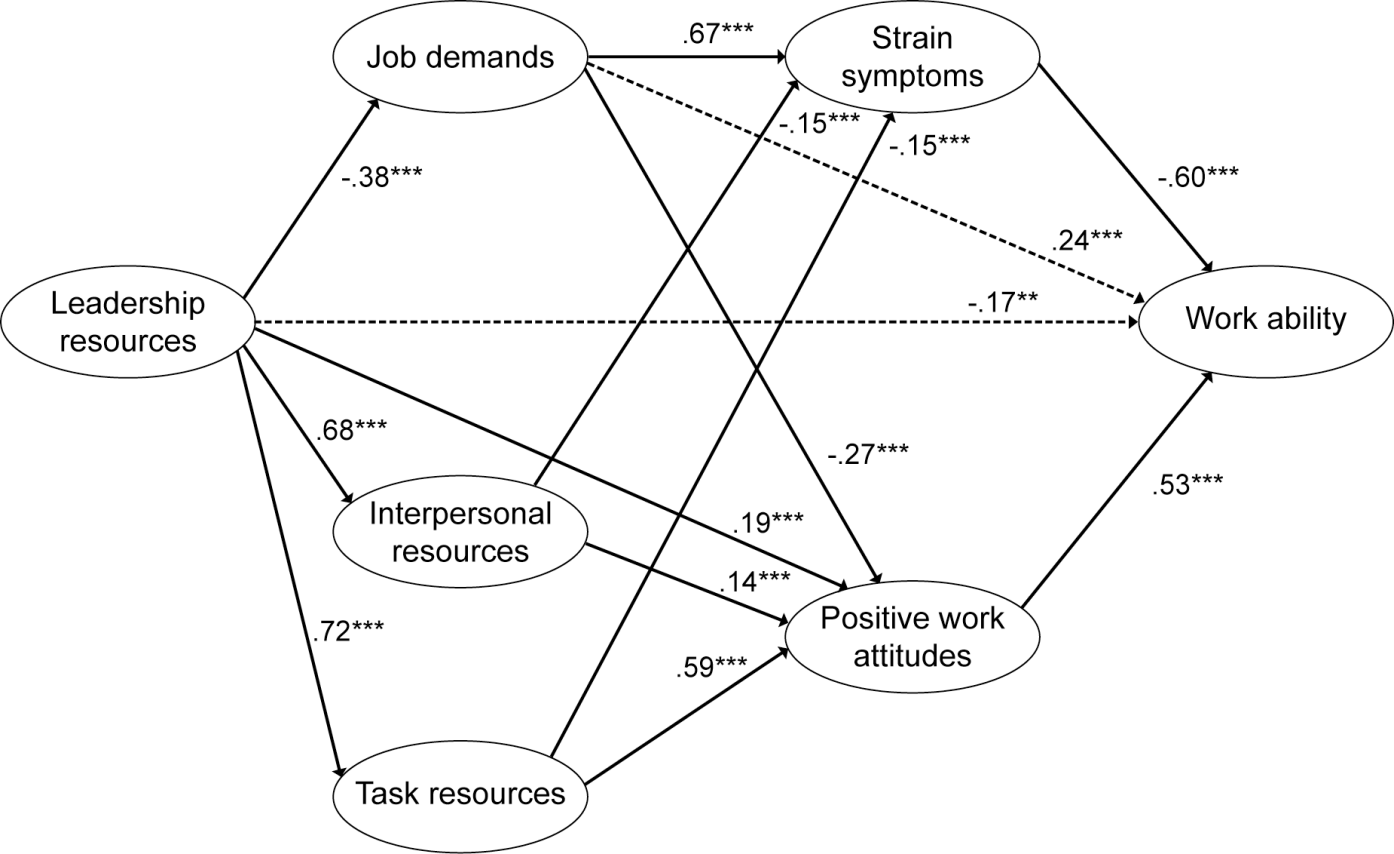
Standardized estimates for the final model (Model E). Note. *** p < .001; ** p < .01;.The dashed paths are due to suppressor effects.

All the model fits are shown by Table 4. Comparing the different models either by using χ^2^ difference test or AIC measures indicated that model D had the best fit. Removing the three non-significant paths from this partially mediated model gave the final model E (significant paths are presented in Fig 3).

**Table 4:** Fit statistics for the study models (n = 1247).

Model	Model description	χ2	df	CFI	TLI	RMSEA	Model comparisons	AIC	Δ χ2	Δ df
**Model A**	Fully mediated model	1822.184	287	0.909	0.897	0.065		1950.184		
**Model B**	Model A plus direct paths from Leadership to second set of mediators	1775.234	284	0.912	0.899	0.065	B vs. A	1909.234	46.950***	3
**Model C**	Model B plus direct paths from first set of mediators to work ability	1765.202	281	0.912	0.898	0.066	C vs. A C vs. B	1905.202	56.982*** 10.032*	6 3
**Model D**	Model C plus a direct path from Job Demands to Positive Work Attitudes	1631.692	280	0.920	0.907	0.062	D vs. A D vs. B D vs. C	1773.692	190.492*** 143.542*** 133.510***	7 4 1
**Model E (Final Model)**	Model D without insignificant paths	1635.540	283	0.920	0.908	0.062	E vs. A	1771.540	186.644***	4

*** p < .001;

** p < .01;

* p < .05

We found two unexpected associations: Leadership had a direct, weakly negative effect on WA (β was–.17, p<.01). Job Demands had a weakly positive effect on WA (β was .24, p<.001). Theoretically, the signs of these relationships should have been reversed, and according to the correlation table, they are (Table 3). Because of the complexity of the model, we suspected that these results could be due to suppressor effects. Indeed, by removing the paths from Job Demands to Strain Symptoms and from Strain Symptoms to WA, the relationship between Job Demands and WA became non-significant, and removing the paths from Leadership to Task and Interpersonal Resources the relationship between Leadership and WA turned non-significant.

To investigate the robustness of our final model (Fig 3), we investigated the indirect effects in the model. The results indicated that Leadership had indirect effects on Strain Symptoms, Positive Work attitudes, and on WA, and similarly that Job Demands, Task Resources and Interpersonal Resources had indirect effects on WA (Table 5). All in all, the results lend support to our extension of the theoretically based JD-R model and for the relationships between different scales included in the COPSOQ instrument.

**Table 5:** Indirect effects in final model using bootstrapping.

Indirect effects	Standardized coefficients	SE	Lower	Upper	p
**Leadership Resources to** **Positive Work Attitudes**	0.63	0.05	0.54	0.74	<0.001
**Leadership Resources to** **Strain Indicators**	–0.54	0.05	–0.65	–0.44	<0.001
**Leadership Resources to** **Workability**	0.62	0.07	0.48	0.75	<0.001
**Interpersonal Resources to** **Workability**	0.17	0.05	0.09	0.29	<0.001
**Task Resources to** **Workability**	0.40	0.14	0.17	0.70	0.002
**Job Demands to** **Workability**	–0.52	0.08	–0.68	–0.38	<0.001

## Discussion

This study contributed to the literature by showing that: A) The scale characteristics were satisfactory and the COPSOQ instrument could be integrated in the JD-R framework; B) Job resources arising from leadership may be a driver of the two processes included in the JD-R model; and C) Both the health impairment and motivational processes were associated with WA, and the results suggested that leadership may impact WA, in particularly by securing task resources.

The internal reliability measured by Cronbach’s alpha was at an adequate level (0.70–0.95 [61]) for all scales except for *Role Conflicts*. This corresponds with findings from Denmark [6] and it has previously been argued that some items in COPSOQ, including items in *Role Conflicts*, can be regarded as causal indicators rather than effect indicators and are thereby not necessarily inter-correlated [17]. Floor and ceiling effects were on an acceptable level for all scales. The highest ceiling effects were seen for *Meaning in Work*, *Role Clarity* and *Social Community at Work*, which corresponds to results from the Spanish validation study [9].

The overall pattern of associations are in line with results from other COPSOQ studies regarding direction of association between WA and job demands, job resources, strain indicators and job satisfaction [22–26]. While the concurrent and convergent validity of COPSOQ has been explored previously, the present study adds a confirmatory approach based on the theoretical reasoning of the JD-R Model. Investigating the scales in such an overall network of expectations supports the nomological validity of the instrument [68, 69].

Previous research in relation to the JD-R model has shown that WA can be impacted negatively by the health impairment process [70], and positively by the motivational process [71]. Also, relationships between demands, resources and WA has been studied, but to our best knowledge, the present study is the first to investigate both the mediating processes of the JD-R model simultaneously in relation to WA as an outcome.

Our results corroborate the relevance of distinguishing different kinds of resources, as suggested by Demerouti and Bakker [39]. The way leaders exert their leadership affects work characteristics and thereby indirectly the wellbeing as well as the stress level among employees [43, 72, 73]. Schaufeli has previously found the effect of engaging leadership on commitment, employability, self-rated performance and performance behaviour to be mainly mediated through the two paths of the JD-R model [43]. In accordance with this, our results indicated that leadership resources can function as a trigger for both processes of the JD-R model with WA as outcome. Leadership was most strongly associated with resources in both studies, indicating that leadership motivates work more than decreasing demands on the employees. However, while Schaufeli [43] found a direct association between leadership and performance-related outcomes including employability, we did not find a corresponding direct effect of leadership on WA. This may indicate that different mechanisms operate depending on the nature of the outcome.

We found a negative association between demands and positive work attitudes which is not theoretically expected in the JD-R model. This can be understood on the basis that human service work is based on a moral commitment [74]. Therefore, high work pressure may impact the opportunities for delivering good quality of care, which is essential for achieving the intrinsic rewards from patient interaction [75].

### Implications

COPSOQ is a comprehensive instrument including a large number of scales. Understanding the interrelationship between the scales in terms of the JD-R model may facilitate communication with practitioners in their efforts to understand and translate survey results into workplace interventions. Despite much being known about the role of work environment for health, motivational and organizational outcomes, it has proven to be difficult to implement this knowledge in organizational development. Understanding complex issues and theories is essential for a successful transferal from workplace surveys to concrete changes. The overall model is good in the respect that it can be helpful in e.g. training managers and other stakeholders in understanding the two processes and how they interrelate with what is actually mapped in a workplace survey.

The results suggest that a relevant strategy could be promoting a leadership which can improve WA through its effects on job resources and demands. Securing task resources such as opportunities for development, influence on the work situation and having clarity about what is expected in the job seems especially relevant for obtaining positive work attitudes understood as job satisfaction, commitment and engagement. Still, the relative importance of different kind of resources might be contextually dependent. Therefore, further testing is needed for better understanding the role of different kinds of resources and their internal relationships as well as their respective importance for the motivational and the health deteriorating processes. In particular, further research is needed to establish the relationships in a longitudinal perspective and in different contexts.

Also, the results contribute to the applicability of COPSOQ for future research within the framework of the JD-R model. The JD-R model posits that the relevant types of demands and resources vary according to the setting and occupations under study. While this specificity is an advantage as regards relevance of the operationalization, it also reduces the opportunities for investigating the relative importance of factors and their interplay across occupational groups or organizational forms. This kind of knowledge is needed for risk management and organizational development in following up on results. A way forward in addressing the trade-off between the need for generic and tailored instruments could be to include relevant scales from COPSOQ in future studies based on the JD-R framework. This could contribute to research on the roles of similar demands and resources in different settings/occupations and thus provide new knowledge concerning, for example, in which situation a demand becomes challenging in contrast to hindering, or the relative importance of various kind of resources [76].

### Strengths and limitations

Our study is innovative as it is the first time that most of the COPSOQ scales are tested in one comprehensive model simultaneously in relation to WA. However, some strengths and limitations exist, in particular as regards the study population and the design of the study.

The response rate of our study was high and the internal non-response low compared to previous COPSOQ validation studies [6, 8–10]. The findings concerning psychometric characteristics and associations of the study model are in accordance with results from previous studies on the instrument. This supports the reliability and validity of the Swedish version of COPSOQ for use in a broader context than dentistry. In addition, the fact that the study is theoretically based and parts of the model have been tested earlier provides some support for generalisation of the overall pattern of associations also to other national versions of the COPSOQ instrument.

However, the cross-sectional design and the use of self-reported data only constitute a clear limitation of our study as it increases the risk of confounding and reverse causality.

The data collected from individuals were nested in workplaces and organizations actualizing a potential need for applying multilevel analyses. However, for the vast majority of scales a rather modest variance was attributed to the workplace level (ICC(1)< 0,10) and the design effect below 2, which is considered to be a relevant cut-off for when clustering in the data needs to be taken into account [77, 78].

Still, the results should be interpreted with caution and be tested in other populations preferable using a longitudinal design integrating register data and multilevel methods if applicable.

## Conclusion

The overall findings of the present study supported the reliability and construct validity of the Swedish version of COPSOQ II tested in a structural equation model based on an extended JD-R model and with workability as outcome.

## Supporting information

S1 Dataset SPSS data file. (SAV)
